# Reducing parents’ vaccination hesitancy: a new educational training model in Turkish sample

**DOI:** 10.3389/fpubh.2026.1808239

**Published:** 2026-05-08

**Authors:** Gizem Solmaz Tümsek, Zehra Baykal Akmeşe

**Affiliations:** Department of Midwifery, Institute of Health Sciences, Ege University, Izmir, Türkiye

**Keywords:** vaccine hesitancy, vaccination, neonate, NICU, midwife, training, health education, vaccine opposition

## Abstract

**Purpose:**

This study aimed to evaluate the effect of the New Education Model (NETM) on vaccine hesitancy among parents of infants hospitalized in a Neonatal Intensive Care Unit (NICU).

**Design and methods:**

This study was conducted using a one-group pretest–posttest quasi-experimental design. The intervention consisted of a structured, two-hour NETM program delivered through interactive sessions. Data were collected using the Personal Information Form (PIF) and the Vaccine Opposition Scale (VOS).

**Results:**

Following the NETM intervention, a statistically significant decrease was observed in mean VOS scores from pre-test to post-test (*p* < 0.001). Significant improvements were also identified across all subscales, including reductions in attitudes legitimizing vaccine hesitancy, strategies to avoid vaccination, and overall hesitancy levels.

**Conclusion:**

The findings suggest that the NETM intervention is associated with improvements in vaccine-related attitudes among parents in the NICU setting.

**Practice implications:**

The NETM represents a structured and feasible educational approach that may support healthcare professionals, particularly nurses and midwives, in addressing vaccine hesitancy through individualized and interactive communication strategies.

## Introduction

Vaccination is a cornerstone of both individual and population health, providing effective protection against a wide range of infectious diseases from early life onward (38). Immunization begins in the neonatal period and continues throughout childhood in accordance with national vaccination schedules, forming a continuous and cumulative protective process [(1): 627; (2): 384]. Neonatal vaccination plays a critical role in initiating early immune responses against severe infectious diseases such as diphtheria, pertussis, tetanus, and hepatitis B, while also contributing to broader public health through the development of herd immunity [(43): 1292; (3): 304; (4): 3795; (5): 1] (41). High vaccination coverage not only protects vaccinated individuals but also indirectly safeguards vulnerable populations, including those who cannot be immunized due to medical conditions. The effectiveness of early-life vaccination strategies has been clearly demonstrated in the control of highly contagious diseases such as measles and poliomyelitis [(6): 1203; (7): 191].

Despite these well-established benefits, vaccine hesitancy has emerged as a growing global public health challenge, affecting immunization uptake across diverse socio-economic and cultural contexts [(8): 1; (9): 6649; (10): 1562–1,568]. Vaccine hesitancy is a complex and multidimensional phenomenon influenced by misinformation, trust deficits, and socio-cultural factors, particularly among parents responsible for childhood vaccination decisions (35, 42).

Low health literacy and insufficient access to reliable information are among the key determinants of vaccine hesitancy [(11): 104–241; (12): 590]. Although educational interventions have been shown to improve vaccine-related knowledge and attitudes, conventional approaches often rely on passive information delivery and may be insufficient to address deeply rooted cognitive biases or misinformation [(13): 137–140; (9)]. However, there remains a significant gap in theory-driven, structured, and context-specific educational interventions designed for high-risk clinical environments such as the Neonatal Intensive Care Unit (NICU). Existing approaches frequently lack individualized and interactive components capable of addressing both the cognitive and emotional dimensions of vaccine hesitancy (29, 30, 32, 33). Moreover, limited attention has been given to integrating validated attitudinal measurement tools within intervention-based models to assess changes in parental perceptions in real-world clinical settings (42).

To address this gap, the present study employs the New Education Model (NETM), an original and structured educational intervention grounded in Social Cognitive Theory and the Information Processing Model. Unlike conventional educational approaches, NETM adopts an interactive, individualized, and feedback-oriented framework that actively engages parents in the learning process and targets the deconstruction of vaccine-related misinformation (18–21). In this study, the Vaccine Opposition Scale (VOS), a validated instrument for assessing parental attitudes toward vaccination, was used to evaluate pre and post-intervention changes in vaccine hesitancy (45).

The Neonatal Intensive Care Unit (NICU) represents a unique clinical environment in which parents experience heightened emotional vulnerability and increased receptivity to health-related information. Trust-based communication established by nurses and midwives in such settings plays a crucial role in shaping parental attitudes and correcting misconceptions (14–17).

Accordingly, this study aims to evaluate the effectiveness of the NETM-based educational intervention in reducing vaccine hesitancy among parents of infants receiving care in the NICU, thereby contributing to the development of evidence-based clinical practices and strengthening public health strategies aimed at improving vaccination uptake.

## Design and methods

This study was conducted using a one-group pretest-posttest quasi-experimental design to evaluate the effectiveness of the New Education Model (NETM) on vaccine hesitancy. The study was carried out in the Neonatal Intensive Care Unit (NICU) of a major State Children’s Hospital in Izmir, Turkey, between March 2025 and June 2025.

The study was designed as a one-group pretest–posttest quasi-experimental study. The process began with the screening of eligible parents in the NICU, followed by the administration of the Personal Information Form (PIF) and the Vaccine Opposition Scale (VOS) as baseline (pre-test) measurements. Subsequently, the structured NETM intervention was delivered individually to participants, with a mean duration of 115 min. Following the intervention, post-test assessments were conducted using the same measurement tools to evaluate changes in vaccine hesitancy.

### Study population and sample

**Figure 1:**
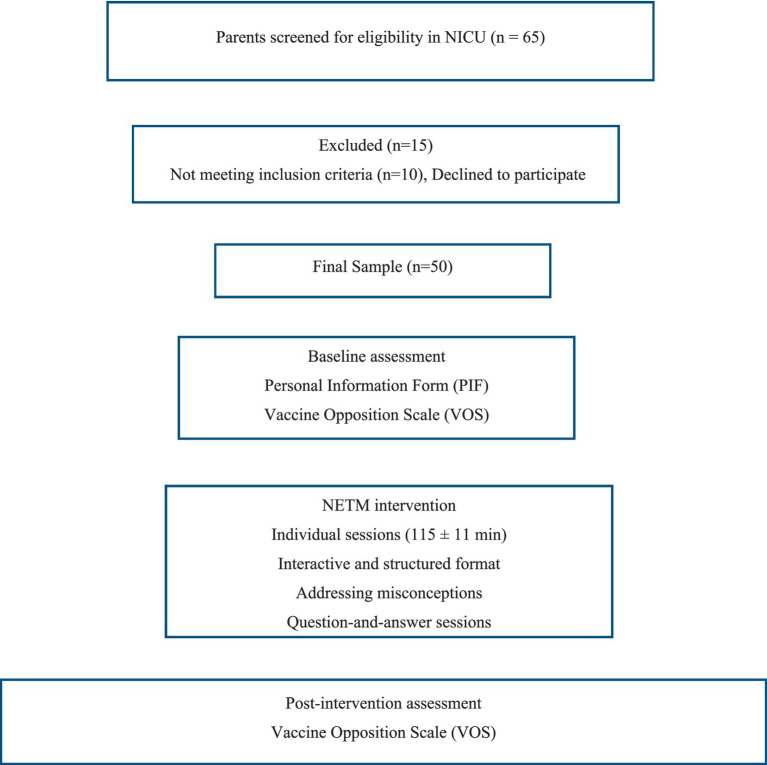
Study flowchart.

The study population consisted of parents of infants hospitalized in the Neonatal Intensive Care Unit (NICU). Participants were recruited using a convenience sampling method. The inclusion criteria were as follows: (i) being the primary caregiver of an infant hospitalized in the NICU for at least 48 h, (ii) being aged 18 years or older, (iii) being literate in Turkish, and (iv) self-reporting vaccine hesitancy or refusal. The exclusion criteria included parents with severe psychiatric or cognitive impairments, those whose infants had medical contraindications to vaccination (e.g., immunodeficiency), and those who had previously participated in a similar educational intervention. The sample size was calculated using G*Power software (version 3.1.9.7). Based on a medium effect size (*d* = 0.50), a significance level (*α*) of 0.05, and a statistical power (1 − *β*) of 0.95, the minimum required sample size was determined to be 45 participants. To account for potential attrition, a total of 50 parents were recruited, and all participants completed the study (Figure 1).

### Setting

The study was conducted in a Level III NICU with a capacity of 40 beds, providing advanced neonatal care. To ensure privacy and minimize external distractions, all educational interventions and data collection procedures were carried out in a designated “Parent Education Room” within the hospital.

### The new education model (NETM) intervention

The New Education Model (NETM) is a structured, theory-informed, and interactive educational intervention designed to address the underlying determinants of vaccine hesitancy through evidence-based communication strategies. The content of the intervention was developed based on current literature (18–21, 44) and subsequently reviewed and validated by a panel of five experts, including two professor neonatologists, one specialist neonatologist, and two midwives. The intervention was delivered individually to each participant by a trained researcher midwife and a specialist neonatologist experienced in vaccine counseling. It was conducted in a designated training room within the hospital to ensure privacy and minimize external distractions. The intervention was administered immediately following the pre-test assessment, with each session lasting approximately 115 ± 11 min.

The educational content included the scientific foundations of vaccination, the neonatal immunization schedule in accordance with national guidelines, and the identification and deconstruction of common vaccine-related misconceptions. The intervention was delivered using visual and written materials supported by peer-reviewed evidence. An individualized and interactive approach was adopted, including one-on-one discussions and structured question-and-answer sessions, allowing participants to actively engage with the content and express their concerns.

### Data collection tools

Data were collected using a Personal Information Form (PIF) and the Vaccine Opposition Scale (VOS).

The Personal Information Form was developed by the researchers to obtain descriptive data on participants’ sociodemographic characteristics and vaccination-related background. The form consisted of 20 items, including variables such as age, gender, education level, income status, and previous vaccination history.

The Vaccine Opposition Scale (VOS), developed by Kılınçarslan et al. (45), is a 21-item, 5-point Likert-type scale designed to assess attitudes toward vaccination. The scale comprises four subscales: Benefits, Antagonism, Alternative Solutions, and Justification of Hesitancy. Higher scores indicate greater levels of vaccine hesitancy.

In the present study, the internal consistency of the scale was evaluated, and Cronbach’s alpha coefficients for the subscales ranged between 0.859 and 0.928, indicating high reliability.

### Data analysis

Data were analyzed using IBM SPSS Statistics (version 23.0; IBM Corp., Armonk, NY, United States). The normality of the data distribution was assessed using the Shapiro–Wilk test. Descriptive statistics were used to summarize participant characteristics, including means and standard deviations for continuous variables and frequencies and percentages for categorical variables. To evaluate changes in vaccine hesitancy scores before and after the intervention within the same participants, the Paired Samples t-test was applied, as the data met the assumption of normal distribution. Effect sizes were calculated using Cohen’s d to assess the magnitude of the intervention effect, with values of 0.5 and 0.8 interpreted as moderate and large effects, respectively. All statistical tests were two-tailed, and the level of statistical significance was set at *p* < 0.05.

### Ethical considerations

Ethical approval for this study was obtained from the Ege University Faculty of Medicine Clinical Research Ethics Committee (Date: 20.02.2025; Decision No: 25–2.1-T/99). Institutional permission was granted by İzmir Dr. Behçet Uz Children’s Diseases and Surgery Training and Research Hospital. Additionally, official approval was obtained from the İzmir Provincial Health Directorate following submission of the required documentation. The study was conducted in accordance with the principles of the Declaration of Helsinki. Written informed consent was obtained from all participants prior to data collection.

## Results

The mean age of the participants was 33.0 ± 5.7 years, and 54.0% were female. As presented in Table 1, more than half of the participants (56.0%) were university graduates, and 32.0% were self-employed. The majority of the participants were married (84.0%). Income status, based on self-reported data, indicated that 48.0% of participants perceived their income level as high.

**Table 1:** Distribution of participants’ socio-demographic characteristics (*n* = 50).

Variable	Category	*N*	%
Age Group	20–25	4	8.0
	26–30	14	28.0
	31–35	15	30.0
	36–40	11	22.0
	40+	6	12.0
Gender	Female	27	54.0
	Male	23	46.0
Educational status	Primary school	2	4.0
	Middle school	1	2.0
	High school	13	26.0
	Bachelor’s degree	28	56.0
	Postgraduate degree	6	12.0
Occupation	Worker	11	22.0
	Civil servant	15	30.0
	Self-employed/Tradesman	16	32.0
	Housewife	8	16.0
Marital status	Married	42	84.0
	Divorced	8	16.0
Income level	Very low	1	2.0
	Low	6	12.0
	Medium	14	28.0
	High	24	48.0
	Very high	5	10.0

Regarding vaccination history, 72.0% of the participants reported having been vaccinated previously, with COVID-19 being the most commonly reported vaccine (47.2%). Additionally, 60.0% of parents had vaccinated their other children. Among those who had not (40.0%), the primary reasons included distrust of vaccines (50.0%) and concerns about side effects (15.0%).

### Vaccine perceptions and concerns

Prior to the intervention, nearly half of the participants (48.0%) reported low confidence in vaccine reliability, while 60.0% expressed uncertainty regarding the positive effects of vaccines on infant health. The internet was identified as the primary source of vaccine-related information for 64.0% of participants.

The most commonly reported concerns were potential side effects (56.0%) and vaccine contents (26.0%). Although 40.0% of participants did not support mandatory vaccination policies, all participants (100%) indicated a strong willingness to receive structured, evidence-based information through the NETM.

As presented in Table 1, the mean age of the participants was 33.0 ± 5.7 years, with the highest proportion in the 31–35 age group (30.0%). More than half of the participants were female (54.0%) and held a bachelor’s degree (56.0%). In terms of occupational distribution, participants were predominantly self-employed/tradesmen (32.0%) or civil servants (30.0%). The majority of the participants were married (84.0%).

Regarding income status, nearly half of the participants (48.0%) reported a high income level, whereas 14.0% reported low or very low income levels. Income categories were determined based on predefined ranges provided to participants, and responses were recorded according to these categories.

### Effectiveness of the NETM intervention

The comparison of parents’ mean Vaccine Opposition Scale (VOS) scores before and after the NETM intervention is presented in Table 2.

**Table 2:** Comparison of parents’ mean VOS scores in pre-test, and post-test (*n* = 50).

VOS subscales	Test	Mean +/− SD	95% CI	*t*-value*	*p*-value
Belief in vaccine benefit	Pre-test	11.96 ± 4,44	10.70–13.22	−4.933	<0.001
	Post- test	17.72 ± 4,44	16.46–18.98		<0.001
Vaccine opposition	Pre-test	22.58 ± 5,44	21.03–24.13	−5,784	<0.001
	Post- test	15.54 ± 5,68	13.93–17.15		<0.001
Strategies to avoid vac.	Pre-test	18.06 ± 5,04	16.63–19.49	−5.210	<0.001
	Post- test	12.62 ± 5,47	11.07–14.17		<0.001
Legitimation of hesitancy	Pre-test	15.12 ± 5,23	13.63–16.61	−3.933	<0.001
	Post- test	10.96 ± 4,89	9.57–12.35		<0.001

*p* < 0.05, Paired samples *t*-test was used, and results are presented with 95% confidence intervals.

Statistically significant differences were observed across all four VOS subscales following the intervention (all *p* < 0.001). Specifically, the mean score for the “Belief in Vaccine Benefit” subscale increased from 11.96 ± 4.44 (95% CI: 10.70–13.22) to 17.72 ± 4.44 (95% CI: 16.46–18.98), indicating an improvement in positive perceptions toward vaccination. In contrast, mean scores for “Vaccine Opposition” decreased from 22.58 ± 5.44 to 15.54 ± 5.68, “Strategies to Avoid Vaccination” decreased from 18.06 ± 5.04 to 12.62 ± 5.47, and “Legitimation of Hesitancy” decreased from 15.12 ± 5.23 to 10.96 ± 4.89. These reductions reflect a consistent decline in negative attitudes and avoidance-related behaviors associated with vaccination. Overall, the findings demonstrate that the NETM intervention was associated with significant improvements in vaccine-related attitudes among parents in the NICU setting. Detailed results, including 95% confidence intervals, are presented in Table 2.

## Discussion

In this study, the effectiveness of the New Education Model (NETM) in addressing vaccine hesitancy among parents in a Neonatal Intensive Care Unit (NICU) was evaluated. The findings indicate that a structured, interactive, and individualized educational approach is associated with improvements in parental attitudes and reductions in vaccine hesitancy. Prior to the intervention, a substantial proportion of parents reported low confidence in vaccine reliability and uncertainty regarding the benefits of vaccination for infant health. These findings are consistent with the literature, which identifies concerns about vaccine content and fear of side effects as key drivers of vaccine hesitancy (22, 23, 35, 37, 40).

A notable finding was the high reliance on the internet as a primary source of information, which may increase susceptibility to misinformation and contribute to the formation of negative vaccination attitudes. This is consistent with previous research indicating that digital environments play a significant role in shaping parental perceptions of vaccines (31, 34, 39).

Another key finding of this study was the statistically significant change observed across all subscales of the Vaccine Opposition Scale (VOS) following the NETM intervention. These findings suggest that the NETM approach may be effective in modifying attitudinal components of vaccine hesitancy. Unlike traditional, unidirectional information delivery methods, NETM is grounded in Social Cognitive Theory and the Information Processing Model, which emphasize active engagement and cognitive restructuring.

By prioritizing the deconstruction of specific misconceptions through individualized, one-on-one interactions, the model facilitates personalized risk communication. This interactive format not only addresses informational gaps but also supports trust-building through direct engagement with a healthcare professional. Such approaches have been reported to be more effective than passive informational materials in influencing deeply rooted cognitive biases related to vaccination (26, 27).

The observed increase in the “Belief in Vaccine Benefit” subscale suggests that providing parents with evidence-based, context-specific, and peer-supported information may strengthen perceptions related to social responsibility and herd immunity. This finding is consistent with previous research indicating that interventions addressing the underlying justification of vaccine hesitancy are more likely to produce sustained attitudinal change than those focusing solely on clinical information (21, 28).

In addition, the reduction in scores for the “Vaccine Hesitancy” and “Strategies to Avoid Vaccination” subscales suggests that the NETM approach may be effective in weakening the cognitive frameworks that parents use to justify vaccine refusal. These findings indicate that targeting both informational and cognitive dimensions of hesitancy may play a critical role in shaping more favorable attitudes toward vaccination.

### Limitations

Despite its important findings, this study has several limitations that should be considered when interpreting the results. First, the use of a single-group pretest–posttest quasi-experimental design without a control group limits the ability to attribute the observed changes exclusively to the intervention, as potential external influences cannot be fully ruled out. Second, the relatively small sample size (*n* = 50) and the single-center setting may limit the generalizability of the findings to broader populations. Third, the post-test assessment was conducted immediately after the intervention, which prevents evaluation of the long-term sustainability of the observed attitudinal changes. In addition, the study focused on attitudinal outcomes and did not assess actual vaccination behavior; therefore, it remains unclear whether the observed improvements translate into real-world vaccination uptake.

## Conclusion and practice implications

This study provides robust evidence that the New Education Model (NETM) constitutes an effective and context-sensitive intervention for reducing vaccine hesitancy among parents in the NICU setting. The findings underscore the importance of moving beyond passive information delivery toward structured, individualized, and interactive educational approaches that foster trust and address parental concerns in a responsive manner. Accordingly, healthcare professionals—particularly nurses and midwives—should prioritize one-on-one, engagement-based counseling models to enhance communication quality and facilitate informed decision-making. In addition, given the pervasive influence of digital misinformation, it is imperative to actively guide parents toward reliable, evidence-based online resources, thereby strengthening their digital health literacy and resilience against misleading content (31, 34, 39). At the institutional level, these results highlight the need for integrating standardized vaccine counseling protocols, such as NETM, into routine neonatal care practices, enabling healthcare systems to systematically leverage the NICU as a critical “teachable moment” and ultimately contribute to improved vaccine uptake and broader community immunity.

## Conclusion

In conclusion, this study highlights that parental vaccine hesitancy in the Neonatal Intensive Care Unit (NICU) is a significant public health challenge driven primarily by perceived risks and distrust in vaccine safety. The implementation of the New Education Model (NETM) has proven to be a highly effective intervention, leading to a statistically significant reduction in vaccine hesitancy and an increase in positive attitudes toward immunization.

The findings underscore that while parents may harbor deep-seated concerns—often fueled by unreliable internet sources-they remain highly receptive to structured, evidence-based information provided by healthcare professionals. The success of the NETM suggests that addressing vaccine hesitancy requires moving beyond traditional, passive information transfer. Instead, interactive and individualized counseling models that target the “epistemological” and emotional roots of parental fears are essential for achieving sustainable behavioral change (36).

Based on the findings of the present study, several key implications can be articulated for clinical practice, policy, and future research. First, in clinical settings, it is essential that healthcare professionals—particularly neonatal nurses and midwives—are systematically trained in structured, interactive counseling approaches such as the NETM model, as these facilitate trust-based, empathetic communication and may effectively address vaccine hesitancy among parents. From a policy perspective, institutional protocols should be strengthened to incorporate mandatory, evidence-based vaccine education sessions during the NICU stay, thereby capitalizing on the “teachable moment” characterized by heightened parental receptivity in this critical period. Finally, in terms of future research, there is a clear need for methodologically rigorous studies, particularly randomized controlled trials with extended longitudinal follow-up (e.g., 6–12 months), to evaluate the sustained impact of such educational interventions not only on attitudinal change but also on actual vaccine uptake and broader community-level immunization outcomes.

## Data Availability

The raw data supporting the conclusions of this article will be made available by the authors, without undue reservation.
